# Heteromerization of Ciliary G Protein-Coupled Receptors in the Mouse Brain

**DOI:** 10.1371/journal.pone.0046304

**Published:** 2012-09-27

**Authors:** Jill A. Green, Chen Gu, Kirk Mykytyn

**Affiliations:** 1 Department of Pharmacology, College of Medicine, The Ohio State University, Columbus, Ohio, United States of America; 2 Department of Neuroscience, College of Medicine, The Ohio State University, Columbus, Ohio, United States of America; University of Florida, United States of America

## Abstract

Nearly every cell type in the mammalian body projects from its cell surface a primary cilium that provides important sensory and signaling functions. Defects in the formation or function of primary cilia have been implicated in the pathogenesis of many human developmental disorders and diseases, collectively termed ciliopathies. Most neurons in the brain possess cilia that are enriched for signaling proteins such as G protein-coupled receptors and adenylyl cyclase type 3, suggesting neuronal cilia sense neuromodulators in the brain and contribute to non-synaptic signaling. Indeed, disruption of neuronal cilia or loss of neuronal ciliary signaling proteins is associated with obesity and learning and memory deficits. As the functions of primary cilia are defined by the signaling proteins that localize to the ciliary compartment, identifying the complement of signaling proteins in cilia can provide important insights into their physiological roles. Here we report for the first time that different GPCRs can colocalize within the same cilium. Specifically, we found the ciliary GPCRs, melanin-concentrating hormone receptor 1 (Mchr1) and somatostatin receptor 3 (Sstr3) colocalizing within cilia in multiple mouse brain regions. In addition, we have evidence suggesting Mchr1 and Sstr3 form heteromers. As GPCR heteromerization can affect ligand binding properties as well as downstream signaling, our findings add an additional layer of complexity to neuronal ciliary signaling.

## Introduction

Primary cilia are typically solitary, immotile cellular appendages that coordinate specialized signaling [1], [2], [3]. They extend from nearly all mammalian cell types and are required for proper development and cellular homeostasis [4]. Loss of cilia or ciliary signaling proteins is associated with a group of diseases termed ciliopathies [5]. Due to the ubiquity of cilia and their critical roles in numerous signaling pathways, ciliopathies present with a wide range of clinical features, including cystic kidney disease, retinal degeneration, obesity, skeletal defects, hypogonadism, anosmia, intellectual disability, and brain malformations. Although the precise functions of most primary cilia are unknown, it is clear their functions are defined by the proteins that are targeted to and retained in the ciliary compartment [6]. Thus, identification of the proteins enriched within cilia can lend important insight into their functions.

Most neurons in the mammalian brain possess primary cilia that are enriched for specific signaling proteins, including adenylyl cyclase type 3 (AC3) [7], which converts ATP to cAMP, and the G protein-coupled receptors (GPCRs) somatostatin receptor 3 (Sstr3) [8], serotonin receptor 6 (Htr6) [9], melanin-concentrating hormone receptor 1 (Mchr1) [10], and dopamine receptor 1 (D1) [11]. The colocalization of GPCRs with AC3 in neuronal cilia suggests they mediate cAMP signaling in response to GPCR activation. Yet, the precise functions of GPCRs on neuronal cilia and how they impact neuronal function is unknown. Studies using conditional knockout mice have implicated neuronal cilia in the regulation of feeding behavior. Disruption of cilia specifically on POMC-expressing neurons in the hypothalamus, a region of the brain involved in the regulation of appetite, causes hyperphagia-induced obesity in mice [12]. Mchr1, which is a key regulator of feeding behavior, localizes to neuronal cilia in the hypothalamus of wildtype mice but fails to localize to cilia on neurons lacking proteins mutated in the human obesity syndrome Bardet-Biedl syndrome (BBS) [13]. Neuronal cilia have also been linked to learning and memory. Mice lacking either Sstr3 or AC3 display defective novel object recognition [14], [15]. Although knockout mouse models do not distinguish the contribution of ciliary signaling from non-ciliary signaling, the fact that Sstr3 and AC3 are enriched together in neuronal cilia and disruption of either one gives a similar learning deficit strongly supports a role for neuronal ciliary signaling in learning and memory. Interestingly, BBS proteins are required for Sstr3 ciliary localization [13] and BBS patients commonly display cognitive deficits.

Identification of GPCRs that are enriched in neuronal cilia and characterization of their distribution throughout the brain can provide insight into the functions of these specialized organelles. Here we report, for the first time, two different ciliary GPCRs, Mchr1 and Sstr3, colocalize within the same cilia in multiple brain regions of mice. In addition to colocalizing, we show that Mchr1 and Sstr3 can interact to form heteromers. As GPCR heteromerization can affect ligand binding properties and downstream signaling, our findings add a previously unrecognized layer of complexity to neuronal ciliary signaling.

## Results

### Mchr1 localizes to neuronal cilia throughout the mouse brain

**Figure 1:**
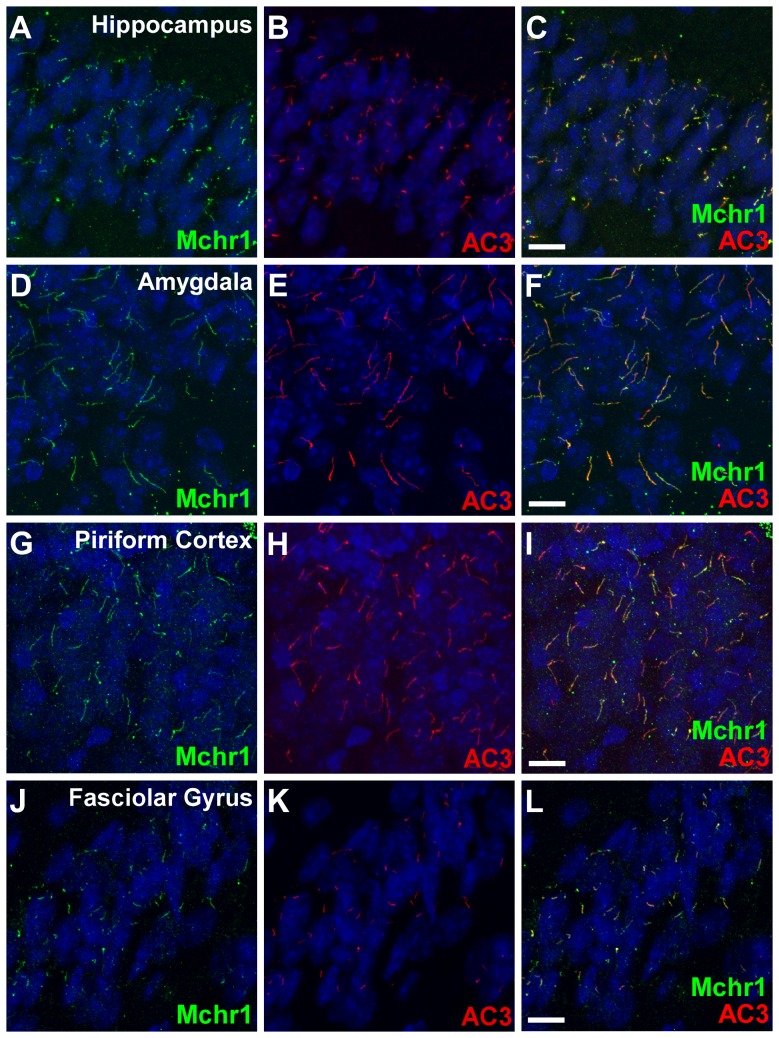
Mchr1 localizes to neuronal cilia throughout the mouse brain. (A–L) Representative images of multiple brain regions in 5–6 week old WT mice showing colabeling for Mchr1 (green) and AC3 (red). Nuclei are stained with DRAQ5 (blue). Labeling for AC3 reveals numerous primary cilia present throughout the CA1 region of the hippocampus (B), amygdala (E), piriform cortex (H), and fasciolar gyrus (K). Labeling for Mchr1 (A, D, G, & J) reveals abundant Mchr1 ciliary localization in each region. Merged images (C, F, I, L) showing colocalization between Mchr1 and AC3. Scale bars represent 10 µm.

Melanin-concentrating hormone (MCH) is a cyclic neuropeptide that has been implicated in a variety of physiological processes such as food intake and energy balance, anxiety, depression, sleep, reward, and cognitive function. In rodents, the physiological functions of MCH are mediated by a single receptor, Mchr1, which shows the highest homology to the somatostatin receptor family [16]. We previously demonstrated Mchr1 ciliary localization in several regions of the mouse brain, including the hypothalamus, striatum, and nucleus accumbens [10], [13], [17]. To better understand Mchr1's function and provide insight into the prevalence of Mchr1 ciliary signaling, we performed a thorough analysis of Mchr1 ciliary localization throughout the mouse brain. Wild type (WT) brain slices were colabeled with antibodies to adenylyl cyclase type 3 (AC3), the canonical neuronal ciliary marker [7], and Mchr1. In addition to the previously described regions, we detected Mchr1-positive cilia in the hippocampus, amygdala, piriform cortex, and fasciolar gyrus (Fig. 1). Overall, Mchr1 ciliary localization is widespread throughout the mouse brain (Table 1), which correlates with the widespread expression pattern of Mchr1 mRNA in the mouse brain [18].

**Table 1:** Distribution of Mchr1 positive cilia in the mouse brain.

Region of the Brain	Relative Immunolabeling
Amygdala	++
Cortex-Motor	+
Cortex-Somatosensory	+
Cortex-Visual	+
Cortex-Auditory	+
Cortex-Piriform	+++
Fasciolar Gyrus	+++
Hippocampus-CA1	+++
Hippocampus-CA2	++
Hippocampus-CA3	+
Hypothalamus	++
Nucleus Accumbens	++++
Olfactory Tubercle	+++
Striatum	++

The relative number of Mchr1-positive cilia in each brain region, normalized to AC3-positive cilia, is designated by: +, sparse distribution of Mchr1-positive cilia; ++, moderate distribution of Mchr1-positive cilia; +++, extensive distribution of Mchr1-positive cilia; and ++++, highest detection of Mchr1-positive cilia.

### Mchr1 and Sstr3 colocalize to a subset of neuronal cilia in specific brain regions

**Figure 2:**
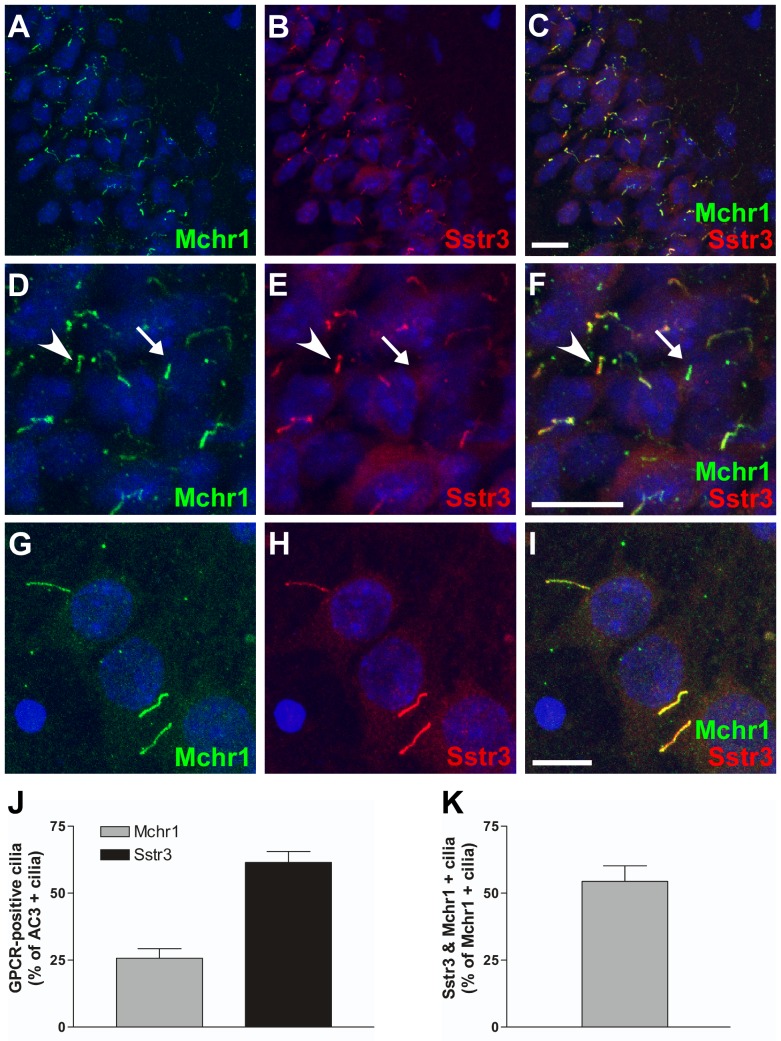
Mchr1 and Sstr3 colocalize in a subset of hippocampal neuronal cilia. (A–F) Representative image of the CA1 region of the hippocampus from an adult WT mouse colabeled for Mchr1 (green) and Sstr3 (red). Nuclei are stained with DRAQ5 (blue). Labeling for Mchr1 (A) and Sstr3 (B) reveals the presence of Mchr1- and Sstr3-positive cilia in the hippocampus. Merged image (C) shows colocalization of Mchr1 and Sstr3 on a subset of neuronal cilia. Zoomed in image (D–F) reveals a subset of neuronal cilia that are positive for Mchr1 only (arrow) and a subset that are positive for both Mchr1 and Sstr3 (arrowhead). (G–I) Colabeling of day 7 WT hippocampal neurons with antibodies to Mchr1 (green) and Sstr3 (red). Nuclei are stained with DRAQ5 (blue). Merged image (I) shows Mchr1 and Sstr3 colocalization within the same cilium *in vitro*. (J) Quantification of day 7 WT hippocampal neurons colabeled for Mchr1 and AC3 or Sstr3 and AC3 reveals that Mchr1localizes to 25.73±3.53% (n = 239) of AC3-positive cilia and Sstr3 localizes to 61.46±4.12% (n = 221) of AC3-positive cilia. (K) Graphical representation of the quantification of day 7 WT hippocampal neurons colabeled for Mchr1 and Sstr3 shows Sstr3 colocalizes to 54.43±5.78% (n = 67) of Mchr1-positive cilia. Scale bars represent 10 µm.

In the hippocampus, Mchr1 ciliary localization was limited to the CA fields. Mchr1-positive cilia were most abundant in the CA1 region with reduced numbers in the CA2 region and very few in the CA3 region. As somatostatin receptor 3 (Sstr3) localizes to cilia throughout the hippocampus, including the CA1 and CA2 regions [8], we tested whether Mchr1 and Sstr3 ever localize within the same cilium. Colabeling brain sections with anti-Mchr1 and anti-Sstr3 antibodies revealed numerous cilia within the hippocampus that were positive for both Mchr1 and Sstr3 (Fig. 2A–F), with the majority of Mchr1/Sstr3 colocalization located in the CA1 and CA2 regions. This is the first time the presence of different GPCRs within the same cilium has been reported.

To quantify Mchr1/Sstr3 colocalization, we prepared primary neuronal cultures from the hippocampus of P0 mice. After 7 days in culture the neurons were fixed and colabeled with antibodies against Mchr1 and AC3 or Sstr3 and AC3. In hippocampal neuronal cultures, approximately 25% of the AC3-positive cilia were Mchr1-positive (Fig. 2J). Sstr3 ciliary localization was much more frequent with approximately 61% of AC3-positive cilia positive for Sstr3 (Fig. 2J). We then colabeled the hippocampal neuronal cultures with antibodies against Mchr1 and Sstr3 to quantify the number of cilia positive for both ciliary GPCRs (Fig. 2G–I). Approximately 54% of Mchr1-positive cilia were also positive for Sstr3 (Figure 2K). Thus, Mchr1 and Sstr3 colocalize to the majority of Mchr1-positive cilia in hippocampal cultures and this finding reflects what was observed in colabeled brain slices. Overall, our results indicate there are at least three different populations of neuronal cilia in the hippocampus; cilia that are positive for Sstr3, cilia that are positive for Mchr1, and cilia that are positive for both Sstr3 and Mchr1.

**Figure 3:**
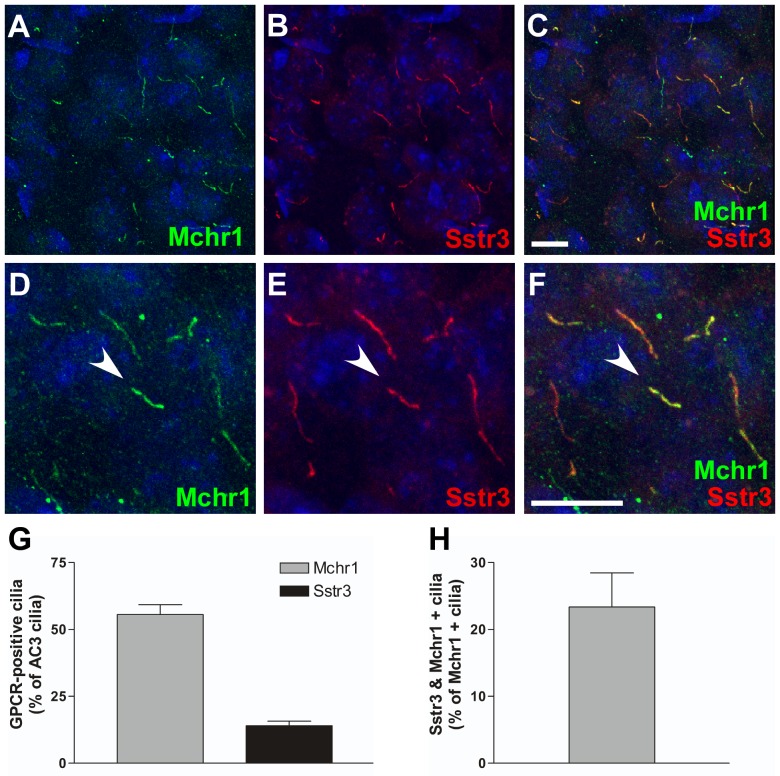
Mchr1 and Sstr3 colocalize in a subset of piriform cortical neuronal cilia. (A–F) Representative image of the piriform cortex from an adult WT mouse colabeled for Mchr1 (green) and Sstr3 (red). Nuclei are stained with DRAQ5 (blue). Labeling for Mchr1 (A) and Sstr3 (B) reveals the presence of Mchr1- and Sstr3-positive cilia in the piriform cortex. Merged image (C) shows colocalization of Mchr1 and Sstr3 on a subset of neuronal cilia. Zoomed in image (D–F) reveals a subset of neuronal cilia that are positive for both Mchr1 and Sstr3 (arrowhead). (G) Quantification of day 7 WT piriform cortical neurons colabeled for Mchr1 and AC3 or Sstr3 and AC3 reveals that Mchr1 localizes to 55.62±3.63% (n = 186) of AC3-positive cilia and Sstr3 localizes to 14.03±1.71% (n = 159) of AC3-positive cilia. (H) Graphical representation of the quantification of day 7 WT piriform cortical neurons colabeled for Mchr1 and Sstr3 shows Sstr3 colocalizes to 23.36±5.11% (n = 86) of Mchr1-positive cilia. Scale bars represent 10 µm.

**Figure 4:**
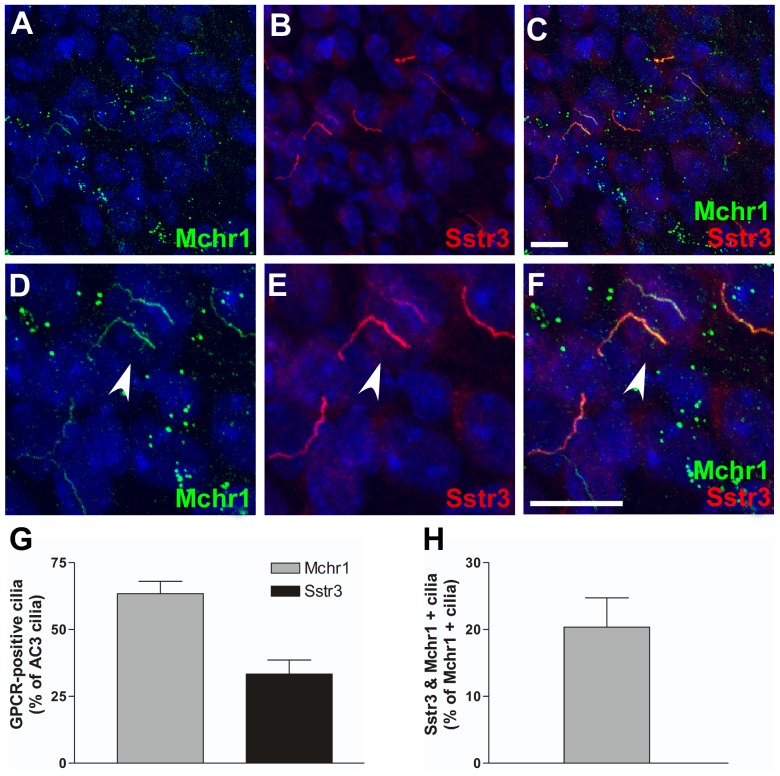
Mchr1 and Sstr3 colocalize in a subset of hypothalamic neuronal cilia. (A–F) Representative image of the hypothalamus from an adult WT mouse colabeled for Mchr1 (green) and Sstr3 (red). Nuclei are stained with DRAQ5 (blue). Labeling for Mchr1 (A) and Sstr3 (B) reveals the presence of Mchr1- and Sstr3-positive cilia in the hypothalamus. Merged image (C) shows colocalization of Mchr1 and Sstr3 on a subset of neuronal cilia. Zoomed in image (D–F) reveals a subset of neuronal cilia that are positive for both Mchr1 and Sstr3 (arrowhead). (G) Quantification of day 7 WT hypothalamic neurons colabeled for Mchr1 and AC3 or Sstr3 and AC3 reveals that Mchr1localizes to 63.42±4.59% (n = 108) of AC3-positive cilia and Sstr3 localizes to 33.34±5.24% (n = 129) of AC3-positive cilia. (H) Graphical representation of the quantification of day 7 WT piriform cortex neurons colabeled for Mchr1 and Sstr3 shows Sstr3 colocalizes to 20.36±4.39% (n = 85) of Mchr1-positive cilia. Scale bars represent 10 µm.

Due to the extensive distribution of Mchr1- and Sstr3-positive cilia throughout the mouse brain, we then asked whether colocalization of these ciliary GPCRs was restricted to the hippocampus or whether it occurred in other brain regions. Examination of brain slices colabeled for Mchr1 and Sstr3 revealed colocalization of Mchr1 and Sstr3 in a subset of neuronal cilia in the piriform cortex (Fig. 3) and hypothalamus (Fig. 4), regions of the brain that play important roles in olfaction and feeding behavior.

To quantify Mchr1/Sstr3 colocalization in the piriform cortex and hypothalamus, we prepared neuronal cultures from these regions and colabeled them with antibodies against Mchr1 and AC3 or Sstr3 and AC3. In piriform cortical cultures approximately 55% of AC3-positive cilia were positive for Mchr1 (Fig. 3G). In contrast, only 14% of AC3-positive cilia were positive for Sstr3 (Fig. 3G). It should be noted that the abundance of Sstr3-positive cilia appeared to be higher in the adult brain slices compared to the neuronal cultures, which may reflect changes in Sstr3 ciliary localization throughout the brain during development [19]. In hypothalamic cultures, approximately 63% of AC3-positive cilia were positive for Mchr1 and 33% of AC3-positive cilia were positive for Sstr3 (Fig. 4G). To quantify the number of cilia positive for both ciliary GPCRs, cultured neurons from the piriform cortex and hypothalamus were colabeled with anti-Mchr1 and anti-Sstr3. Approximately 23% of Mchr1-positive cilia in the piriform cortical neuronal cultures and 20% of Mchr1-positive cilia in the hypothalamic neuronal cultures were also positive for Sstr3 (Figs. 3H and 4H). Thus, similar to the hippocampus, our results indicate there are at least three different populations of neuronal cilia in the piriform cortex and hypothalamus.

### Mchr1 and Sstr3 interact

**Figure 5:**
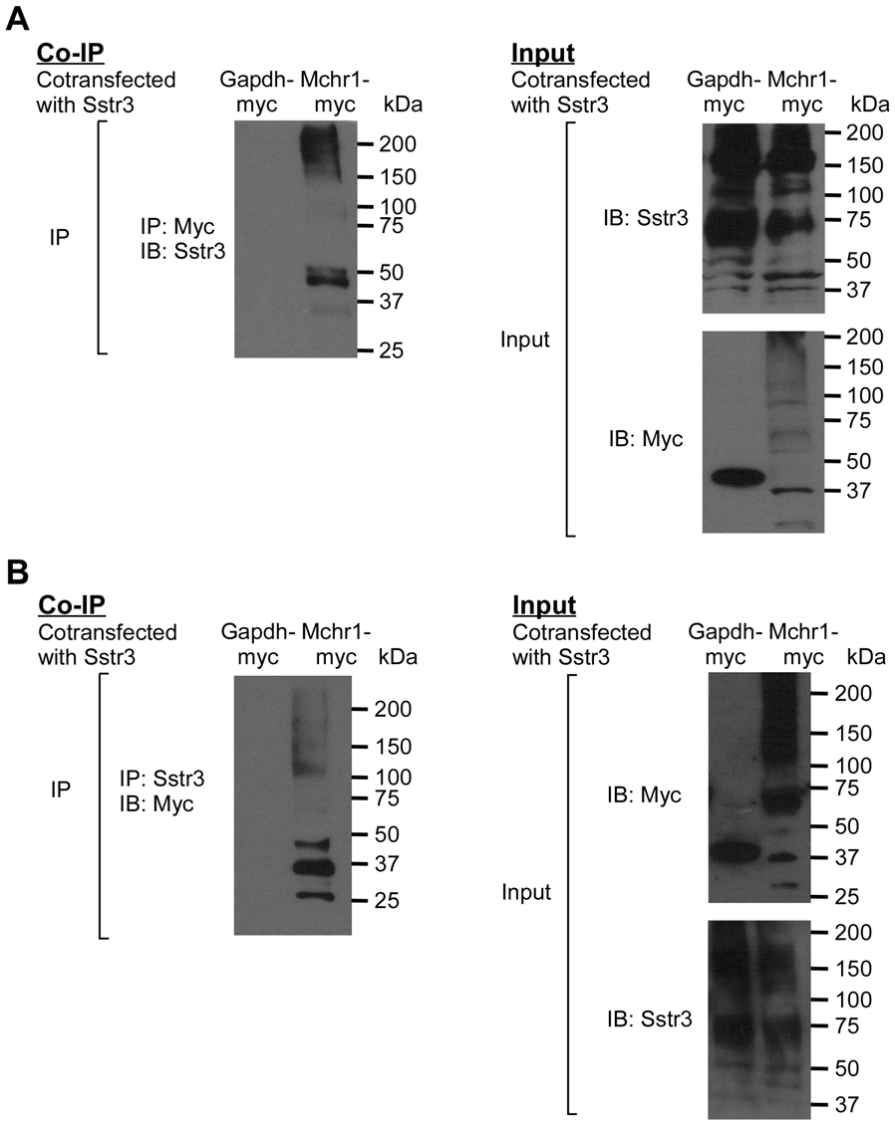
Mchr1 and Sstr3 proteins interact. Sstr3 was co-expressed with myc-tagged glyceraldehyde-3-phosphate dehydrogenase (Gapdh-myc) or myc-tagged Mchr1 (Mchr1-myc) in HEK293T cells. (A) Cell extracts were immunoprecipitated (IP) with an anti-myc antibody. Immunoprecipitates were analyzed by western blotting (IB) with an anti-Sstr3 antibody (left). Note that Sstr3 is immunoprecipitated with Mchr1, as indicated by the 45 kDa band, but not Gapdh. (B) In the reverse experiment, cell extracts were immunoprecipitated (IP) with an anti-Sstr3 antibody and immunoprecipitates were analyzed by western blotting (IB) with an anti-myc antibody (left). Note that Mchr1 is immunoprecipitated with Sstr3, as indicated by the 37 kDa band, but not Gapdh. The input, confirming expression of each protein, is also shown (right). Sstr3 appears as a 45 kDa band, which agrees with its predicted size, and 75 kDa and 150 kDa bands, which may represent higher order oligomers. Gapdh and Mchr1 are observed as 39 kDa and 37 kDa bands, respectively. The expression pattern of GPCRs often results in a multi-band pattern due to various oligomeric combinations and post translational modifications.

Mchr1 and Sstr3 on the same cilium may signal independently of one another or they may interact and form heteromers that could generate a signal unique from their individual components. To begin to address this question, we tested whether Mchr1 and Sst3 interact and form a heteromer. HEK293T cells were transiently cotransfected with constructs encoding Sstr3 and either myc-tagged Mchr1 or myc-tagged Gapdh, as a negative control. Lysates were precipitated with an anti-myc antibody and immunoblotted with an anti-Sstr3 antibody. We found that Sstr3 co-immunoprecipitated with Mchr1 but not Gapdh (Fig. 5A). Performing the reverse experiment confirmed Mchr1 is co-immunoprecipitated with Sstr3 but not Gapdh (Fig. 5B). Together, these results indicate the interaction between the two ciliary GPCRs is specific.

**Figure 6:**
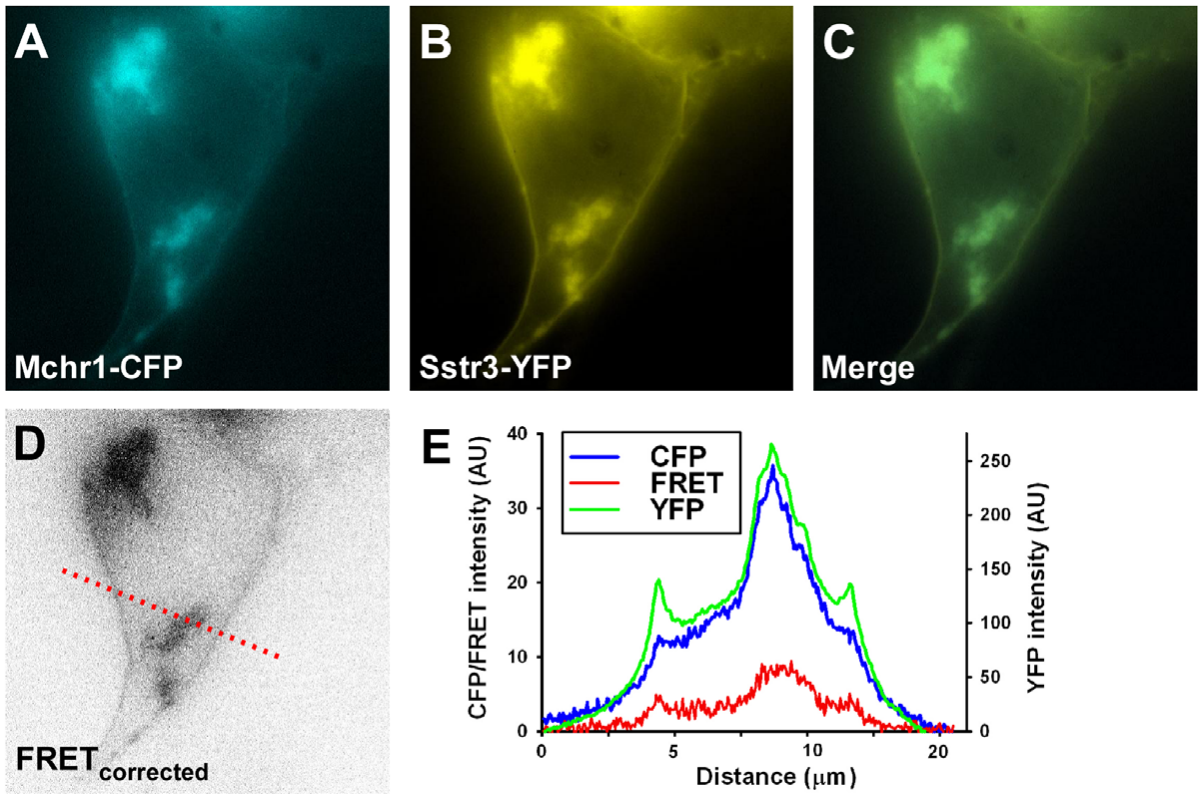
Mchr1 and Sstr3 interact in live cells. HEK293T cells were transiently co-transfected with constructs encoding CFP-tagged Mchr1 and YFP-tagged Sstr3. Mchr1-CFP and Sstr3-YFP colocalize at the plasma membrane and in intracellular compartments (A–C). Robust FRET signals are observed between Mchr1-CFP and Sstr3-YFP localizing in intracellular compartments and moderate FRET signals are observed between Mchr1-CFP and Sstr3-YFP localizing in the cell membrane (D). The fluorescence intensity profile along the red-dotted line within the FRET_corrected_ image is shown (E). The intensity scale for CFP (blue) and FRET_corrected_ (red) are on the left. The intensity scale for YFP (green) is on the right. AU = Arbitrary Unit.

To further validate the interaction between Mchr1 and Sstr3, we performed fluorescence resonance energy transfer (FRET) analysis in live HEK293T cells expressing pECFP-Mchr1 and pEYFP-Sstr3. Some HEK293T cells possessed a cilium upon which the receptors colocalized. However, we excluded the cilium from our FRET analysis as it is possible that colocalization of the receptors within the confines of a cilium could generate an artefactual FRET signal. The two GPCRs highly colocalized (Fig. 6A–C) and produced significant FRET signals (Fig. 6D, E) throughout the plasma membrane as well as some intracellular compartments (30 out of 40 cells). The colocalization and FRET signal at the plasma membrane of HEK293T cells is an indication that Mchr1 and Sstr3 can interact and potentially heteromerize in the ciliary membrane. The observed colocalization and FRET signal in intracellular compartments may be due to intracellular accumulation of overexpressed proteins. Alternatively, this may suggest the two GPCRs are trafficked together in the secretory pathway or recycled together in the endocytic pathway. Taken together with our co-immunoprecipitation data these results suggest heterologously expressed Mchr1 and Sstr3 can heteromerize in HEK293T cells.

**Figure 7:**
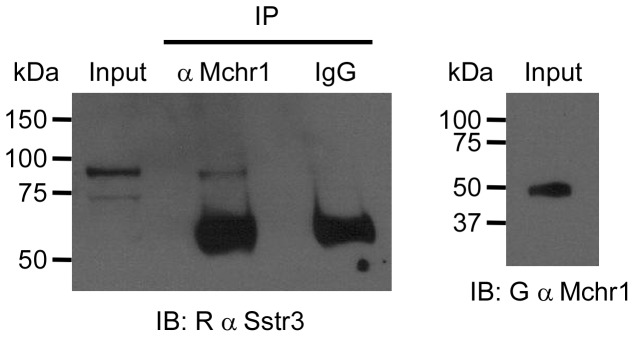
Mchr1 and Sstr3 interact in mouse hippocampal lysate. Membrane protein enriched cell lysate from the hippocampus of 5 week old adult WT mice was immunoprecipitated (IP) with a goat anti-Mchr1 antibody or goat IgG, as a negative control. Immunoprecipitates were analyzed by western blotting (IB) with a rabbit anti-Sstr3 antibody. Note that Sstr3 is immunoprecipitated with Mchr1 but not with the IgG negative control. The input probed with anti-Sstr3 (left) or anti-Mchr1 (right), confirms the expression of Sstr3 and Mchr1. The ∼55 kDa bands in the IP lanes may be IgG heavy chain that is detected due to cross-reactivity of the secondary antibody.

To determine if endogenous Mchr1 and Sstr3 interact within the mouse brain, we performed a co-immunoprecipitation from hippocampal cell lysate that was enriched for membrane proteins. Cell lysate was immunoprecipitated with goat anti-Mchr1 or goat IgG, as a negative control, and then immunoblotted with rabbit anti-Sstr3. We found that Sstr3 specifically co-immunoprecipitated with Mchr1 (Fig. 7), indicating endogenous Mchr1 and Sstr3 can form heteromers within the brains of adult mice.

## Discussion

Our results show Mchr1 localizes to neuronal cilia throughout the mouse brain, including the hypothalamus, striatum, hippocampus, amygdala, piriform cortex, fasciolar gyrus and nucleus accumbens. In addition, subsets of Mchr1-positive cilia in the hippocampus, piriform cortex, and hypothalamus are also positive for Sstr3. This is the first time two different GPCRs have been shown to colocalize within the same cilium.

We further demonstrate Mchr1 and Sstr3 can interact via co-immunoprecipitation and FRET analysis, suggesting they form heteromers. This is a significant finding as heteromerization can have important functional consequences on GPCR signaling, including changes in ligand binding properties, G protein coupling, and/or receptor desensitization and internalization [20]. Heteromerization can have synergistic or antagonistic effects on signaling. D3 heteromerization with D1 increases the affinity of dopamine for D1 and potentiates D1 stimulation of adenylyl cyclase [21], [22]. Conversely, the heterodimer formed between Sstr2a and Sstr3 retains Sstr2a signaling while inactivating the Sstr3 subunit, thereby resulting in diminished Sstr3-like activity [23]. Importantly, signaling modulation is not restricted to GPCRs within the same family. In the context of the D2-Sstr5 heteromer, agonist occupation of D2 increases Sstr5's affinity for SST and potentiates signaling [24]. GPCR heteromers may also generate a completely unique signal due to changes in G protein coupling. Dopamine Receptor 1 (D1) can couple to Gαs/olf and stimulate the production of cAMP through AC. In contrast, Dopamine Receptor 2 (D2) inhibits cAMP production by coupling to Gαi. However, D1–D2 heteromers couple to Gαq to elicit a Ca^2+^ signal [25], [26]. Further studies are required to determine how Mchr1-Sstr3 heteromerization affects ligand binding, G protein coupling, and/or desensitization and internalization.

It is intriguing there is significant colocalization of Mchr1 and Sstr3 in cilia in the hippocampus. The MCH and SST systems have both been implicated in learning and memory. Specifically, MCH infusion into the rat hippocampus modulates memory [27] and Mchr1 knockout mice demonstrate impaired long-term synaptic plasticity in hippocampal neurons [28], [29]. Sstr3 ciliary localization is also abundant in the hippocampus and Sstr3 is required for novel object recognition [14]. Together, these results implicate hippocampal ciliary signaling in learning and memory and it is tempting to speculate that altered ciliary signaling in the hippocampus may be the basis of cognitive deficits in some ciliopathies. Further studies are required to precisely determine the effects of Mchr1/Sstr3 colocalization on ciliary signaling in the hippocampus and their effects on learning and memory.

Mchr1/Sstr3 ciliary colocalization was also detected in the piriform cortex and hypothalamus. These two brain regions are thought to play an important role in olfaction and feeding behavior. MCH is an important regulator of feeding behavior and elicits an orexigenic effect [30]. Somatostatin signaling has also been shown to affect food intake. Selective central activation of Sstr2 increases food intake in rats [31] and pretreatment of rats with an Sstr3-specific agonist significantly reduces leptin-mediated suppression of food intake [32]. It will be interesting to determine whether Mchr1/Sstr3 colocalization correlates with appetite regulating neurons within the hypothalamus, suggesting a direct role for Mchr1-Sstr3 heteromers in feeding.

Quantification of Mchr1/Sstr3 colocalization *in vitro* reveals differences across regions. In the hippocampus, the majority of Mchr1-positive cilia are positive for Sstr3, whereas in the piriform cortex and hypothalamus the minority of Mchr1-positive cilia are positive for Sstr3. However, our quantification was performed on day 7 neurons. In the case of Sstr3, it is known that the relative abundance of ciliary localization is dynamic and varies with the age of the animal [19]. Thus, it is likely the ratios of Mchr1 and Sstr3 ciliary colocalization change during development and aging. This has been observed with D1 and D2 heteromers. Specifically, the relative levels of signaling from D1–D2 heteromers increases in mice with increasing age [26]. It is also likely other GPCRs colocalize with Mchr1 and/or Sstr3 in these and other regions, which would increase the complexity of ciliary signaling even further. Although D1 is a likely candidate, D1 ciliary localization is rarely observed in WT mouse brains, presumably due to high levels of signals stimulating D1 translocation from cilia [11]. D1 ciliary localization is commonly observed on cultured WT neurons and in preliminary studies we have observed ciliary colocalization of D1 and Mchr1 in neuronal cultures (J.G. and K.M. unpublished observations). Thus, Mchr1 and D1 may form heteromers in subsets of neuronal cilia.

Taken together, our findings suggest there may be functional interactions between different ciliary GPCRs within the same neuronal cilium, possibly allowing a specialized signal to be generated. Understanding the functional relevance of ciliary GPCR heteromerization could elucidate the roles of neuronal cilia in non-synaptic signaling as well as provide new therapeutic avenues for ciliopathies.

## Materials and Methods

### Ethics statement

This study was carried out in strict accordance with the recommendations in the Guide for the Care and Use of Laboratory Animals of the National Institutes of Health. The protocol was approved by the Institutional Animal Care and Use Committee of the Ohio State University (Animal Welfare Assurance #A3261-01).

### Brain tissue processing

The mice used in this study were on a FVB background. Five to six week old animals were anesthetized by 0.2 ml/10 g intraperitoneal injection of 2.5% tribromoethanol (Sigma-Aldrich, St. Louis, MO) to prevent pain and suffering, killed by cardiac puncture, and perfused with phosphate-buffered saline (PBS) followed by a 1∶1 mixture of 4% paraformaldehyde(PFA) and Histochoice (Amresco, Solon, OH). The brains were then subjected to a 2 hour post fix in the 2% PFA/Histochoice mixture at 4°C. The brains were cryoprotected in 30% sucrose in PBS for 16–24 hours and sectioned on a freezing microtome at a thickness of 40 µm.

### Cultured neurons and processing

Primary hippocampal, piriform cortex, and hypothalamic neurons were cultured as previously described [33]. Seven days post plating neuronal cultures were fixed and processed for immunofluorescence as previously described [13].

### Immunofluorescence procedures

Immunofluorescent procedures have been previously described [10]. Briefly, brain tissue and cultured neurons were permeabilized with 0.3% Triton X-100 in PBS with 4% donkey serum, 0.02% sodium azide, and 10 mg/ml bovine serum albumin (BSA). Primary antibody incubations were carried out for 16–24 h at 4°C and secondary antibody incubations were carried out for 1 h at room temperature. Brain tissue was washed six times for ten minutes and cultured neurons were washed three times for five minutes with PBS containing 4% donkey serum, 0.02% sodium azide, and 10 mg/ml BSA after primary and secondary antibody incubations. Primary antibodies included rabbit anti-adenylyl cyclase 3 (C-20; Santa Cruz Biotechnology, Santa Cruz, CA), goat anti-melanin-concentrating hormone receptor 1 (C-17; Santa Cruz Biotechnology), goat anti-somatostatin receptor 3 (M-18; Santa Cruz Biotechnology), and rabbit anti-somatostatin receptor 3 (7986; [8]). Secondary antibodies included Alexa Fluor 488-conjucated donkey anti-goat IgG and Alexa Fluor 546-conjucated donkey anti-rabbit IgG (Life Technologies, Grand Island, NY). Nucleic acids were stained with DRAQ5 (Cell Signaling, Danvers, MA). Tissue or coverslips containing neurons were mounted using Immuno-Mount (Thermo Scientific, Pittsburgh, PA). All samples were imaged on a Zeiss LSM 510 laser scanning confocal microscope at the Hunt-Curtis Imaging Facility in the Department of Neuroscience at The Ohio State University. Multiple consecutive focal planes (Z-stack), spaced at approximately 0.43 µm intervals, were captured. For all collected images, the brightness and contrast of each channel was adjusted using the Zeiss LSM Image Browser program.

### Quantification of neuronal populations

Quantitative analysis of neuronal populations was performed on at least three independent coverslips generated from four different animals. For each coverslip, at least five fields were imaged. The number of Mchr1- or Sstr3-positive cilia and the number of AC3-positive cilia in each image were counted by an individual blinded to the labeling condition. The results were expressed as the percentage of AC3-positive cilia showing either Mchr1 or Sstr3 ciliary colocalization. For Mchr1 and Sstr3 colocalization quantification, the number of Mchr1-positive and Sstr3-positive cilia was counted. The results were expressed as the percentage of cilia colocalizing Mchr1 and Sstr3, normalized to the number of Mchr1 positive cilia within the field. All data are expressed as mean ± standard error of the mean (SEM).

### Cell culture and protein isolation

HEK293T cells (ATCC) were maintained in DMEM supplemented with 10% FBS and 1.5 g/L of sodium biocarbonate (Life Technologies). Sstr3 and Mchr1-myc (Sstr3 and Gapdh-myc for negative control) were cotransfected by electroporation into HEK293T cells. After 48 h, cells were lysed in solubilization buffer (20 mM Tris pH 8.0, 150 mM NaCl, 2 mM EDTA, 10% glycerol, 1% NP-40) supplemented with sodium orthovanadate and protease inhibitor cocktail (Roche, Indianapolis, IN). Following a 1 h incubation at 4°C with end-over-end rotation, cell debris and insoluble material were cleared by centrifugation for 20 min at 15,000× g at 4°C. The resulting supernatant containing soluble protein was collected and the concentration was determined by the Bradford assay (Bio-Rad, Richmond, CA, USA).

### Co-immunoprecipitation and western blotting

Soluble protein was precleared at 4°C with rotation for 1 h with protein A-sepharose beads (GE Healthcare, Piscataway, NJ, USA) pre-equilibrated in detergent buffer (1 mMTris pH 7.5, 5 mM NaCl, 1 mM KCl, 1 mM MgCl2, 1% NP-40) supplemented with protease inhibitor cocktail (Roche). For HEK293T co-immunoprecipitation experiments, precleared protein was incubated overnight at 4°C with anti-myc (9E10; Santa Cruz Biotechnology) or goat anti-Sstr3 immobilized to protein A-sepharose beads. For endogenous co-immunoprecipitation experiments, precleared protein was incubated overnight at 4°C with anti-Mchr1. Beads were washed three times with 1× PBS supplemented with protease inhibitor cocktail (Roche). Immunoprecipitated proteins were subjected to 60°C heat for 15 min in SDS sample buffer to elute purified proteins. Purified proteins were analyzed by previously described immuoblotting procedures [11]. Briefly, for HEK293T co-immunoprecipitation experiments membranes were subjected to SDS-PAGE, probed for Sstr3 using goat anti-Sstr3 followed by detection using horseradish peroxidase-conjugated donkey anti-goat IgG (Santa Cruz Biotechnology). Alternatively, membranes were probed for myc using Mouse anti-myc, followed by detection using horseradish peroxidase-conjugated donkey anti-mouse IgG (Santa Cruz Biotechnology). For endogenous co-immunoprecipitation experiments membranes were subjected to SDS-PAGE, probed with rabbit anti-Sstr3 or goat anti-Mchr1, followed by detection by using horseradish peroxidase-conjugated donkey anti-rabbit IgG or donkey anti-goat IgG (Santa Cruz Biotechnology), respectively.

### Fluorescence resonance energy transfer imaging and quantification

Sensitized emission was the strategy used to perform fluorescence resonance energy transfer (FRET) in living HEK293T cells, as previously described [34], [35]. Briefly, HEK293T cells were transiently co-transfected with constructs containing Mchr1 fused at the C-terminus to ECFP and Sstr3 fused at the C-terminus to EYFP. After 48 hours, cells were imaged using three filter sets: (1) CFP filter set (excitation 430/25 nm; emission 470/30 nm); (2) YFP filter set (excitation 500/20 nm; emission 535/20 nm); (3) FRET filter set (excitation 430/25 nm; emission 535/30 nm). A single dichroic mirror (86004BS; Chroma Technology) was used with all three filter channels. The amount of cross-bleeding of the CFP and YFP channels into the FRET filter set was previously determined. The corrected FRET (FRET_corrected_ = FRET_raw_−(0.65×CFP)−(0.015×YFP)) was calculated by MetaMorph software (n = 40). As a positive control, CFP-Kv1.2 and YFP-Kvβ2 produced robust FRET signals. As a negative control, CFP-Kv1.2 and YFP-Kvβ2K235E produced no significant FRET signal [34], [35].

### Isolation of membrane enriched protein

Isolation of membrane enriched protein from mouse brain has been previously described [11]. Protein quantification was determined by a NanoDrop (Thermo).

## References

[1] Berbari NF, O'Connor AK, Haycraft CJ, Yoder BK (2009) The primary cilium as a complex signaling center. Curr Biol 19: R526–535. 19602418 10.1016/j.cub.2009.05.025 PMC2814769

[2] Satir P, Pedersen LB, Christensen ST (2010) The primary cilium at a glance. J Cell Sci 123: 499–503. 20144997 10.1242/jcs.050377 PMC2818190

[3] Veland IR, Awan A, Pedersen LB, Yoder BK, Christensen ST (2009) Primary cilia and signaling pathways in mammalian development, health and disease. Nephron Physiol 111: p39–53. 19276629 10.1159/000208212 PMC2881330

[4] Goetz SC, Anderson KV (2010) The primary cilium: a signalling centre during vertebrate development. Nat Rev Genet 11: 331–344. 20395968 10.1038/nrg2774 PMC3121168

[5] Hildebrandt F, Benzing T, Katsanis N (2011) Ciliopathies. N Engl J Med 364: 1533–1543. 21506742 10.1056/NEJMra1010172 PMC3640822

[6] Green JA, Mykytyn K (2010) Neuronal ciliary signaling in homeostasis and disease. Cell Mol Life Sci 67: 3287–3297. 20544253 10.1007/s00018-010-0425-4 PMC3349968

[7] Bishop GA, Berbari NF, Lewis JS, Mykytyn K (2007) Type III Adenylyl Cyclase Localizes to Primary Cilia throughout the Adult Mouse Brain. J Comp Neurol 505: 562–571. 17924533 10.1002/cne.21510

[8] Handel M, Schulz S, Stanarius A, Schreff M, Erdtmann-Vourliotis M, et al (1999) Selective targeting of somatostatin receptor 3 to neuronal cilia. Neuroscience 89: 909–926. 10199624 10.1016/s0306-4522(98)00354-6

[9] Brailov I, Bancila M, Brisorgueil MJ, Miquel MC, Hamon M, et al (2000) Localization of 5-HT(6) receptors at the plasma membrane of neuronal cilia in the rat brain. Brain Res 872: 271–275. 10924708 10.1016/s0006-8993(00)02519-1

[10] Berbari NF, Johnson AD, Lewis JS, Askwith CC, Mykytyn K (2008) Identification of Ciliary Localization Sequences within the Third Intracellular Loop of G Protein-coupled Receptors. Mol Biol Cell 19: 1540–1547. 18256283 10.1091/mbc.E07-09-0942 PMC2291422

[11] Domire JS, Green JA, Lee KG, Johnson AD, Askwith CC, et al (2011) Dopamine receptor 1 localizes to neuronal cilia in a dynamic process that requires the Bardet-Biedl syndrome proteins. Cell Mol Life Sci 68: 2951–2960. 21152952 10.1007/s00018-010-0603-4 PMC3368249

[12] Davenport JR, Watts AJ, Roper VC, Croyle MJ, van Groen T, et al (2007) Disruption of intraflagellar transport in adult mice leads to obesity and slow-onset cystic kidney disease. Curr Biol 17: 1586–1594. 17825558 10.1016/j.cub.2007.08.034 PMC2084209

[13] Berbari NF, Lewis JS, Bishop GA, Askwith CC, Mykytyn K (2008) Bardet-Biedl syndrome proteins are required for the localization of G protein-coupled receptors to primary cilia. Proc Natl Acad Sci U S A 105: 4242–4246. 18334641 10.1073/pnas.0711027105 PMC2393805

[14] Einstein EB, Patterson CA, Hon BJ, Regan KA, Reddi J, et al (2010) Somatostatin signaling in neuronal cilia is critical for object recognition memory. J Neurosci 30: 4306–4314. 20335466 10.1523/JNEUROSCI.5295-09.2010 PMC3842454

[15] Wang Z, Phan T, Storm DR (2011) The type 3 adenylyl cyclase is required for novel object learning and extinction of contextual memory: role of cAMP signaling in primary cilia. J Neurosci 31: 5557–5561. 21490195 10.1523/JNEUROSCI.6561-10.2011 PMC3091825

[16] Kolakowski LF Jr, Jung BP, Nguyen T, Johnson MP, Lynch KR, et al (1996) Characterization of a human gene related to genes encoding somatostatin receptors. FEBS Lett 398: 253–258. 8977118 10.1016/s0014-5793(96)01160-x

[17] Domire JS, Mykytyn K (2009) Markers for neuronal cilia. Methods Cell Biol 91: 111–121. 20409783 10.1016/S0091-679X(08)91006-2

[18] Kokkotou EG, Tritos NA, Mastaitis JW, Slieker L, Maratos-Flier E (2001) Melanin-concentrating hormone receptor is a target of leptin action in the mouse brain. Endocrinology 142: 680–686. 11159839 10.1210/endo.142.2.7981

[19] Stanic D, Malmgren H, He H, Scott L, Aperia A, et al (2009) Developmental changes in frequency of the ciliary somatostatin receptor 3 protein. Brain Res 1249: 101–112. 18992731 10.1016/j.brainres.2008.10.024

[20] Rozenfeld R, Devi LA (2011) Exploring a role for heteromerization in GPCR signalling specificity. Biochem J 433: 11–18. 21158738 10.1042/BJ20100458 PMC3115900

[21] Fiorentini C, Busi C, Gorruso E, Gotti C, Spano P, et al (2008) Reciprocal regulation of dopamine D1 and D3 receptor function and trafficking by heterodimerization. Mol Pharmacol 74: 59–69. 18424554 10.1124/mol.107.043885

[22] Marcellino D, Ferre S, Casado V, Cortes A, Le Foll B, et al (2008) Identification of dopamine D1–D3 receptor heteromers. Indications for a role of synergistic D1–D3 receptor interactions in the striatum. J Biol Chem 283: 26016–26025. 18644790 10.1074/jbc.M710349200 PMC2533781

[23] Pfeiffer M, Koch T, Schroder H, Klutzny M, Kirscht S, et al (2001) Homo- and heterodimerization of somatostatin receptor subtypes. Inactivation of sst(3) receptor function by heterodimerization with sst(2A). J Biol Chem 276: 14027–14036. 11134004 10.1074/jbc.M006084200

[24] Rocheville M, Lange DC, Kumar U, Patel SC, Patel RC, et al (2000) Receptors for dopamine and somatostatin: formation of hetero-oligomers with enhanced functional activity. Science 288: 154–157. 10753124 10.1126/science.288.5463.154

[25] Lee SP, So CH, Rashid AJ, Varghese G, Cheng R, et al (2004) Dopamine D1 and D2 receptor Co-activation generates a novel phospholipase C-mediated calcium signal. J Biol Chem 279: 35671–35678. 15159403 10.1074/jbc.M401923200

[26] Rashid AJ, So CH, Kong MM, Furtak T, El-Ghundi M, et al (2007) D1–D2 dopamine receptor heterooligomers with unique pharmacology are coupled to rapid activation of Gq/11 in the striatum. Proc Natl Acad Sci U S A 104: 654–659. 17194762 10.1073/pnas.0604049104 PMC1766439

[27] Monzon ME, de Souza MM, Izquierdo LA, Izquierdo I, Barros DM, et al (1999) Melanin-concentrating hormone (MCH) modifies memory retention in rats. Peptides 20: 1517–1519. 10698129 10.1016/s0196-9781(99)00164-3

[28] Pachoud B, Adamantidis A, Ravassard P, Luppi PH, Grisar T, et al (2010) Major impairments of glutamatergic transmission and long-term synaptic plasticity in the hippocampus of mice lacking the melanin-concentrating hormone receptor-1. J Neurophysiol 104: 1417–1425. 20592115 10.1152/jn.01052.2009 PMC4073994

[29] Adamantidis A, Thomas E, Foidart A, Tyhon A, Coumans B, et al (2005) Disrupting the melanin-concentrating hormone receptor 1 in mice leads to cognitive deficits and alterations of NMDA receptor function. Eur J Neurosci 21: 2837–2844. 15926931 10.1111/j.1460-9568.2005.04100.x

[30] Chung S, Parks GS, Lee C, Civelli O (2011) Recent updates on the melanin-concentrating hormone (MCH) and its receptor system: lessons from MCH1R antagonists. J Mol Neurosci 43: 115–121. 20582487 10.1007/s12031-010-9411-4 PMC3018593

[31] Stengel A, Goebel M, Wang L, Rivier J, Kobelt P, et al (2010) Selective central activation of somatostatin receptor 2 increases food intake, grooming behavior and rectal temperature in rats. J Physiol Pharmacol 61: 399–407. 20814067 PMC4040268

[32] Stepanyan Z, Kocharyan A, Behrens M, Koebnick C, Pyrski M, et al (2007) Somatostatin, a negative-regulator of central leptin action in the rat hypothalamus. J Neurochem 100: 468–478. 17083445 10.1111/j.1471-4159.2006.04219.x

[33] Berbari NF, Bishop GA, Askwith CC, Lewis JS, Mykytyn K (2007) Hippocampal neurons possess primary cilia in culture. J Neurosci Res 85: 1095–1100. 17304575 10.1002/jnr.21209

[34] Gu C, Zhou W, Puthenveedu MA, Xu M, Jan YN, et al (2006) The microtubule plus-end tracking protein EB1 is required for Kv1 voltage-gated K+ channel axonal targeting. Neuron 52: 803–816. 17145502 10.1016/j.neuron.2006.10.022

[35] Xu M, Gu Y, Barry J, Gu C (2010) Kinesin I transports tetramerized Kv3 channels through the axon initial segment via direct binding. J Neurosci 30: 15987–16001. 21106837 10.1523/JNEUROSCI.3565-10.2010 PMC2996050

